# Supplementary figures and images for: Evaluation of both exonic and intronic variants for effects on RNA splicing allows for accurate assessment of the effectiveness of precision therapies
Source: PLoS Genet. 2020 Oct 21;16(10):e1009100. doi: 10.1371/journal.pgen.1009100 (PMC7605713; doi:10.1371/journal.pgen.1009100)

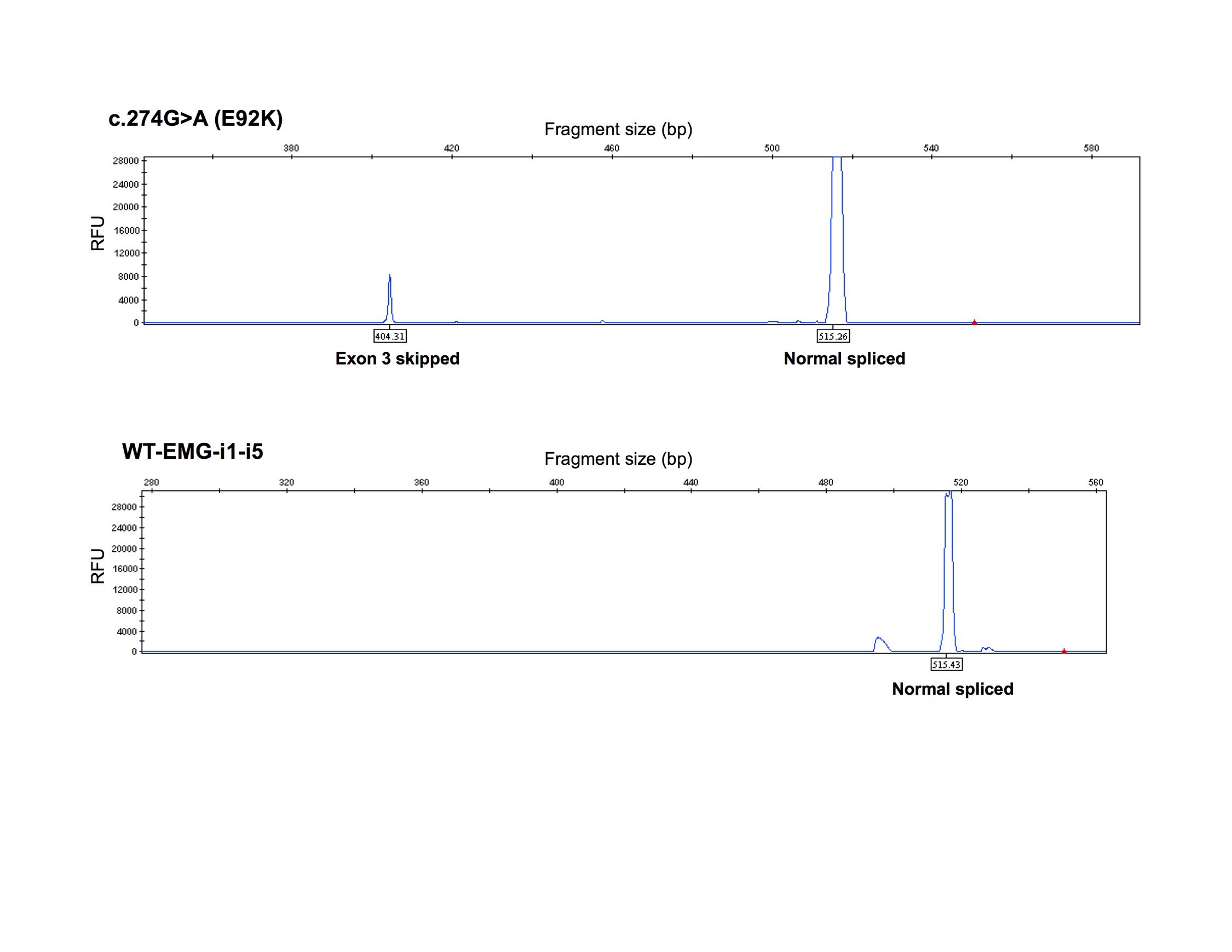

Supplement: S1 Fig — Top panel. Representative raw data for fragment analysis of RT-PCR products from evaluation of splicing in HEK293 cells after transient transfection with EMG_i1-i5 bearing c.274G>A. Results show majority normally spliced transcript and a small fraction of products consistent with the size of exon 3 skipped transcript. Bottom panel. Representative raw data for fragment analysis of RT-PCR products from WT EMG_i1-i5 showing normally spliced transcript. (TIF) [file pgen.1009100.s001.tif]

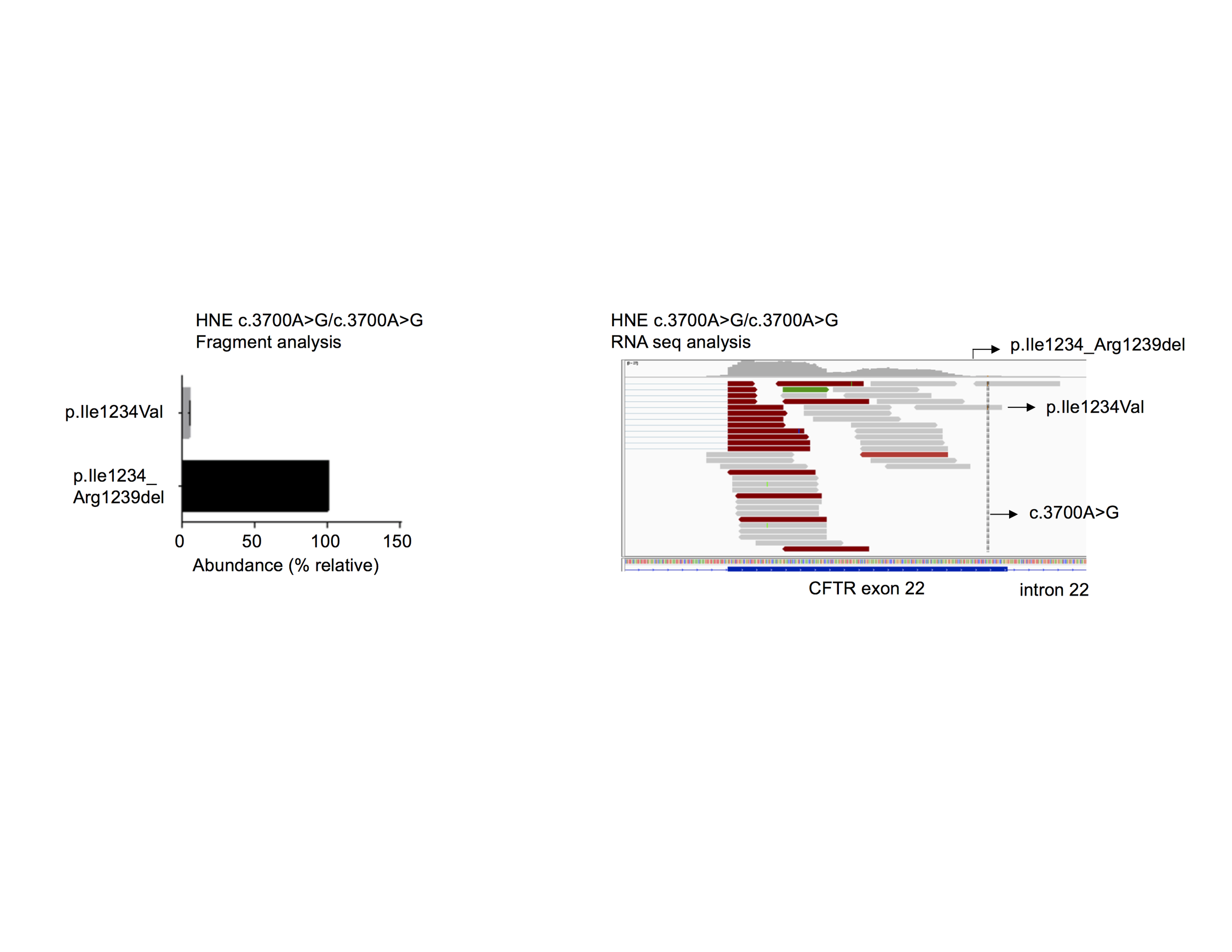

Supplement: S2 Fig — c.3700A>G activation of a cryptic splice site results in production of primarily misspliced transcript. Left panel. Fragment analysis of RT-PCR products shows abundance of normally spliced transcript relative to misspliced transcript. Right panel. Screenshot of IGV view showing RNA sequencing reads mapping to region surrounding c.3700A>G. Reduced coverage is evidence of misspliced product, while one read maps to exon/exon junction indicating some normal splicing. (TIF) [file pgen.1009100.s002.tif]

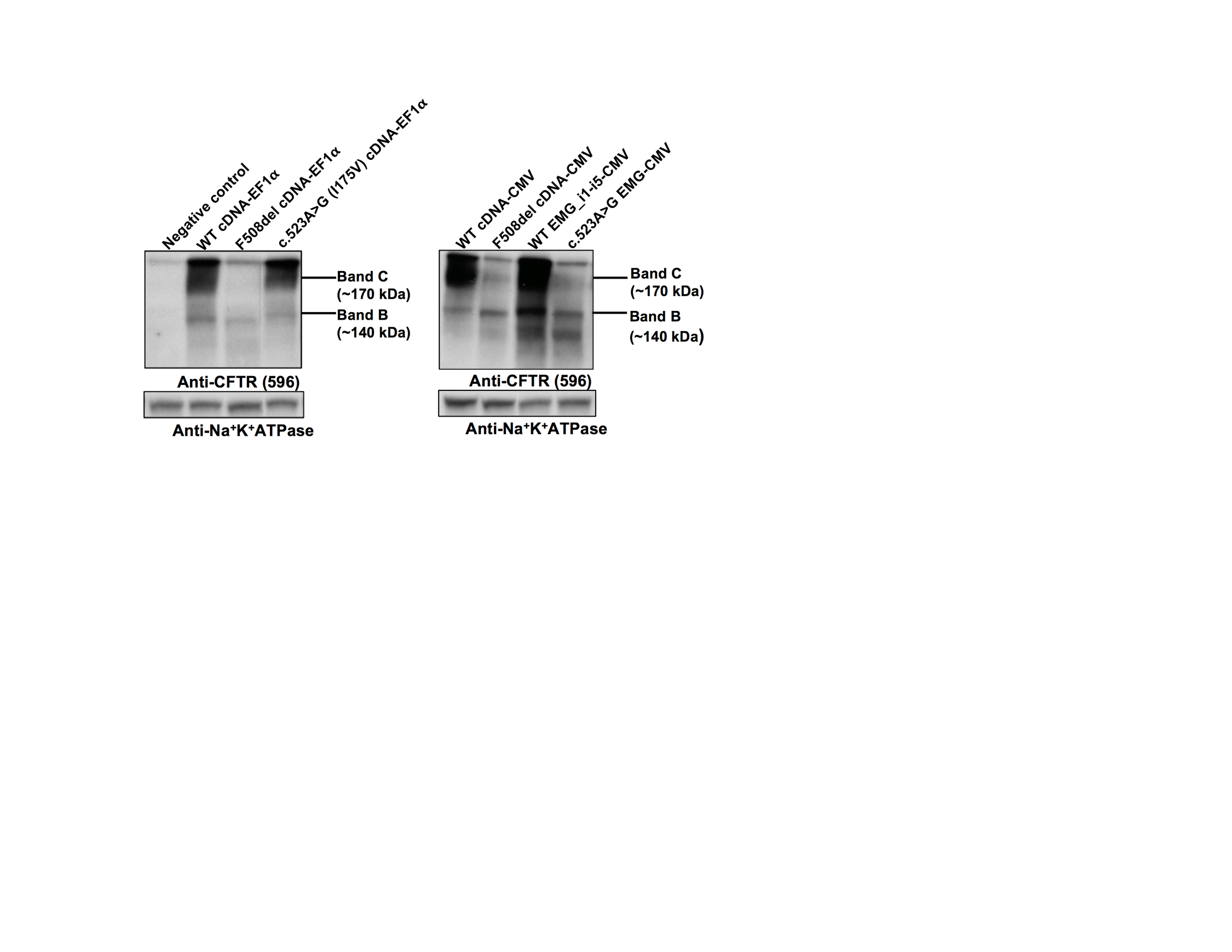

Supplement: S3 Fig — Immunoblot showing CFTR on top, with Na+,K+-ATPase on bottom as a loading control. Lanes from a single blot were reordered and split into two panels to allow for appropriate comparison of matched experimentals and controls. Left panel. All constructs driven by EF1α promoter and express CFTR cDNA. WT and F508del served as controls. Negative control is an empty vector plasmid. Right panel. All constructs driven by CMV promoter and express either CFTR cDNA or CFTR EMG_i1-i5, as indicated. WT cDNA, F508del, WT EMG_i1-i5 served as controls. (TIF) [file pgen.1009100.s003.tif]

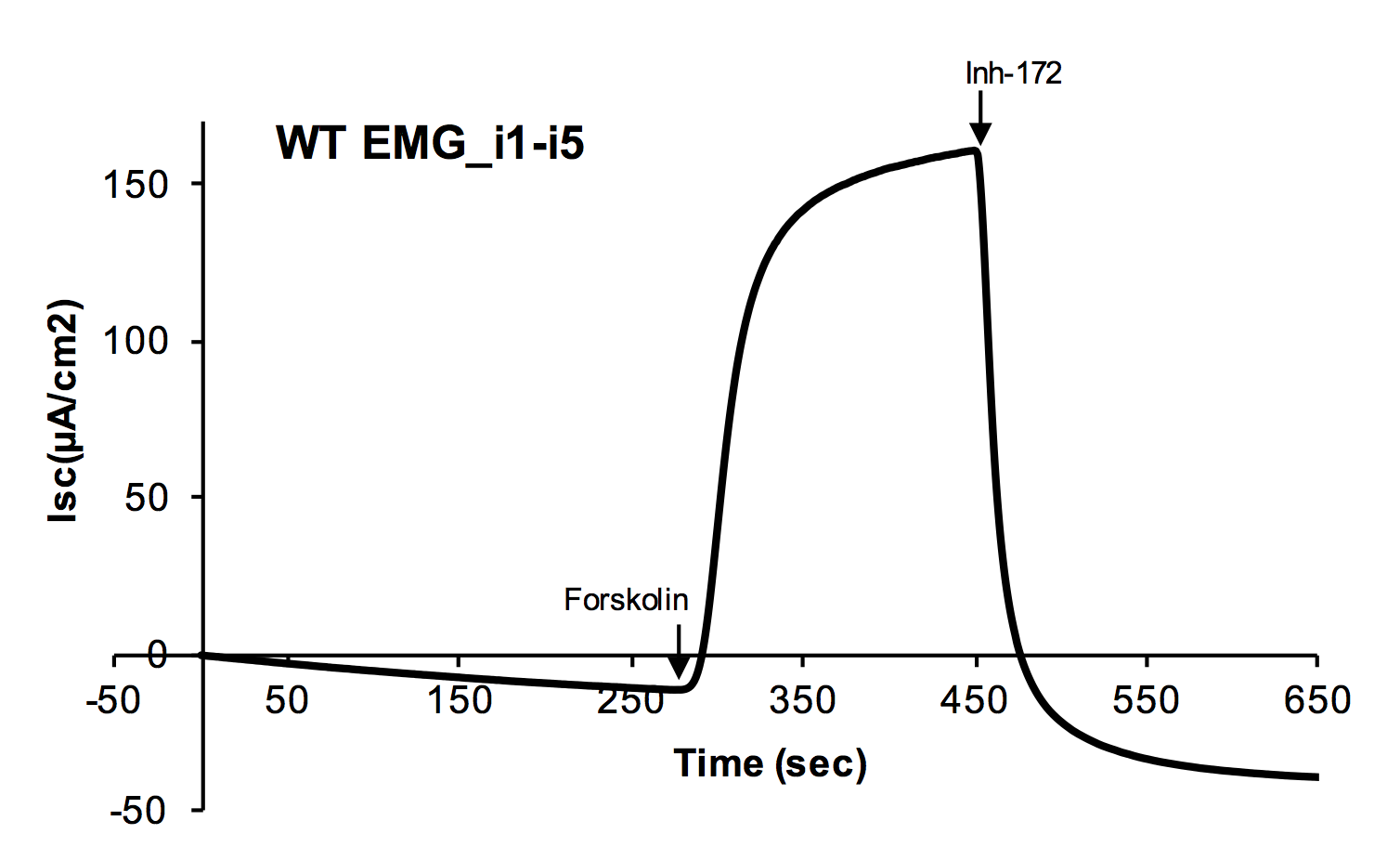

Supplement: S4 Fig — Representative tracing for short circuit current (Isc) assay of CFTR channel function performed on CF bronchial epithelial cells stably expressing the WT EMG_i1-i5 construct. (TIF) [file pgen.1009100.s004.tif]

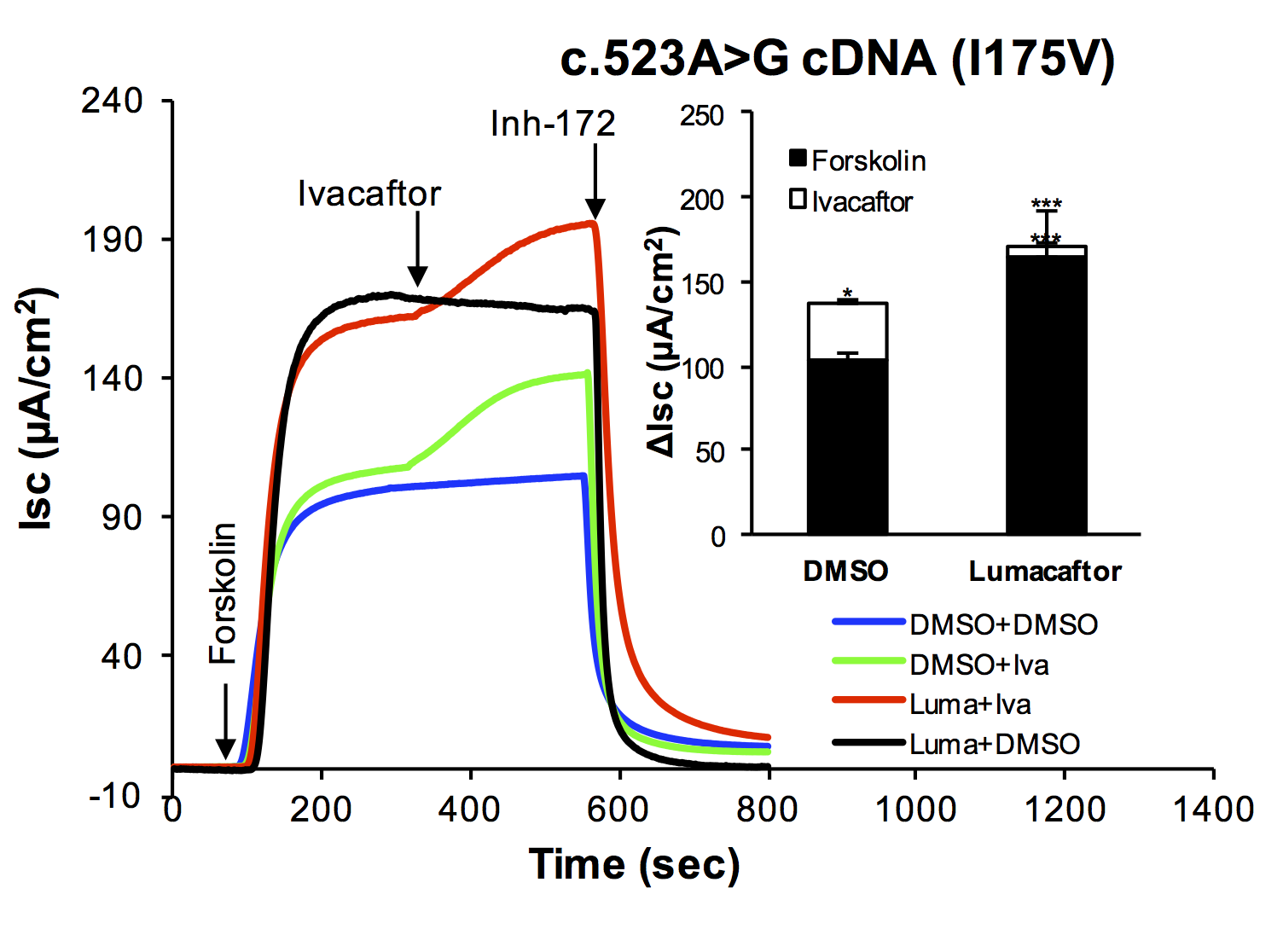

Supplement: S5 Fig — Representative tracings for short circuit current (Isc) assay of CFTR channel function and CFTR modulator response performed on CFBE cells stably expressing the I175V cDNA construct. Inset- Quantification of change in Isc in response to modulators (minimum of three independent measurements per condition). Data shown as mean±SD. p value determined by one-way ANOVA. *** (p≤0.001), *(p≤0.05) when compared to DMSO treated vehicle control. Data underlying graph in this figure reported in S6 Data. (TIF) [file pgen.1009100.s005.tif]

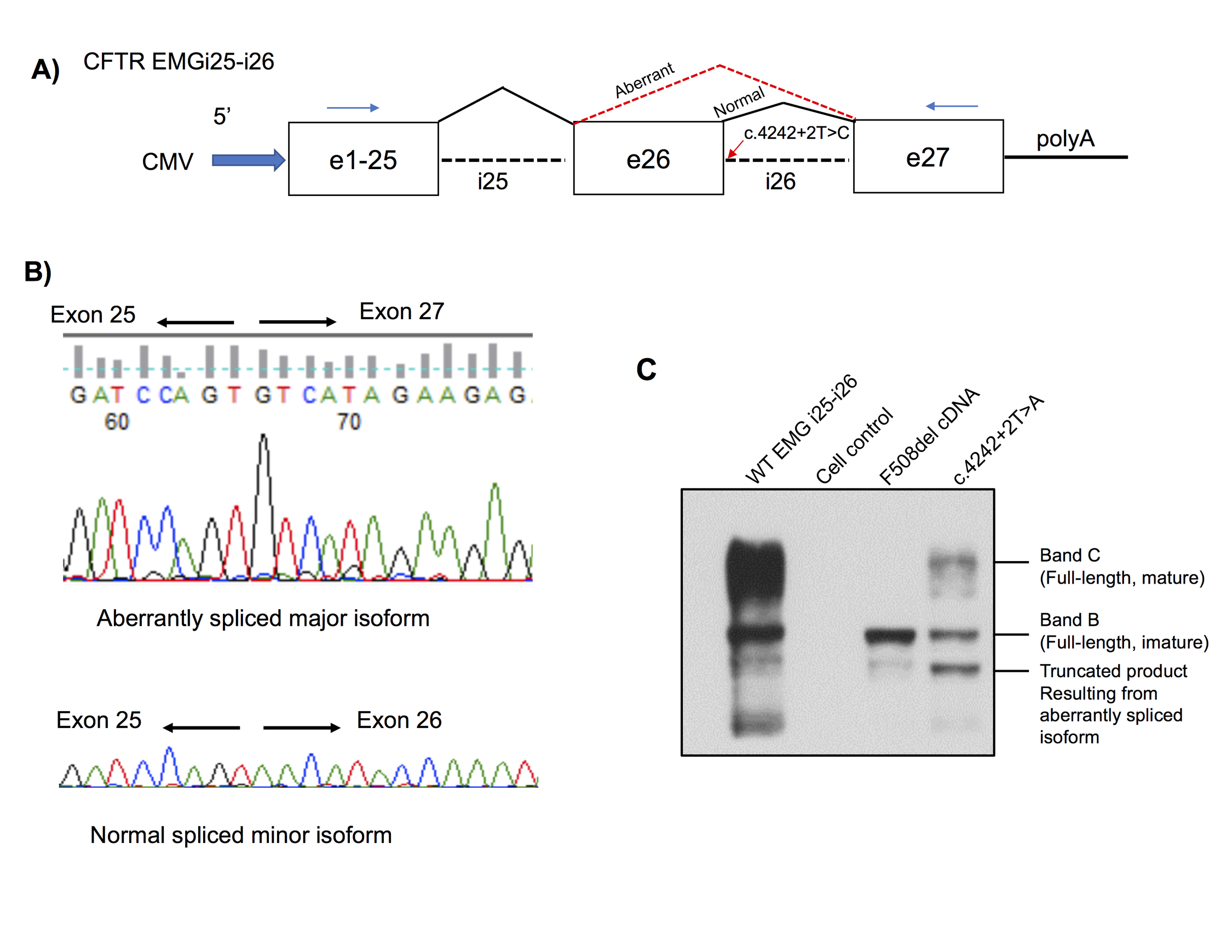

Supplement: S6 Fig — (A) Schematic illustration of the Expression minigene i25-i26 and location of the splice-site variant. Full-length introns 25 and 26 were inserted into pcDNA5FRT-CFTR cDNA construct to create the expression minigene. Variant c.4242+2T>C was created by site directed mutagenesis. Two isoforms (aberrant and normal indicated on the labels) were observed in HEK293Flp cells by transient transfection of the EMG harboring the c.4242+2T>C variant. (B) Sanger sequencing confirmed skipping of exon 26 in the aberrant spliced isoform resulting in frameshift and introduction of premature termination codon. The second minor isoform confirmed normal splicing. (C) Immunoblotting of the protein lysates collected from HEK293Flp cells transfected with either WT EMG_i25-26, F508del cDNA or c.4242+2T>C. Non-transfected parental cells served as control. CFTR antibody 596 (CFFT) was used to probe for CFTR protein production. Residual levels of full-length mature and immature protein products denoted as band C and B respectively were produced by c.4242+2T>C variant due to normal splicing. A lower sized truncated CFTR protein fragment resulting from aberrantly spliced isoform was also observed. (TIF) [file pgen.1009100.s006.tif]

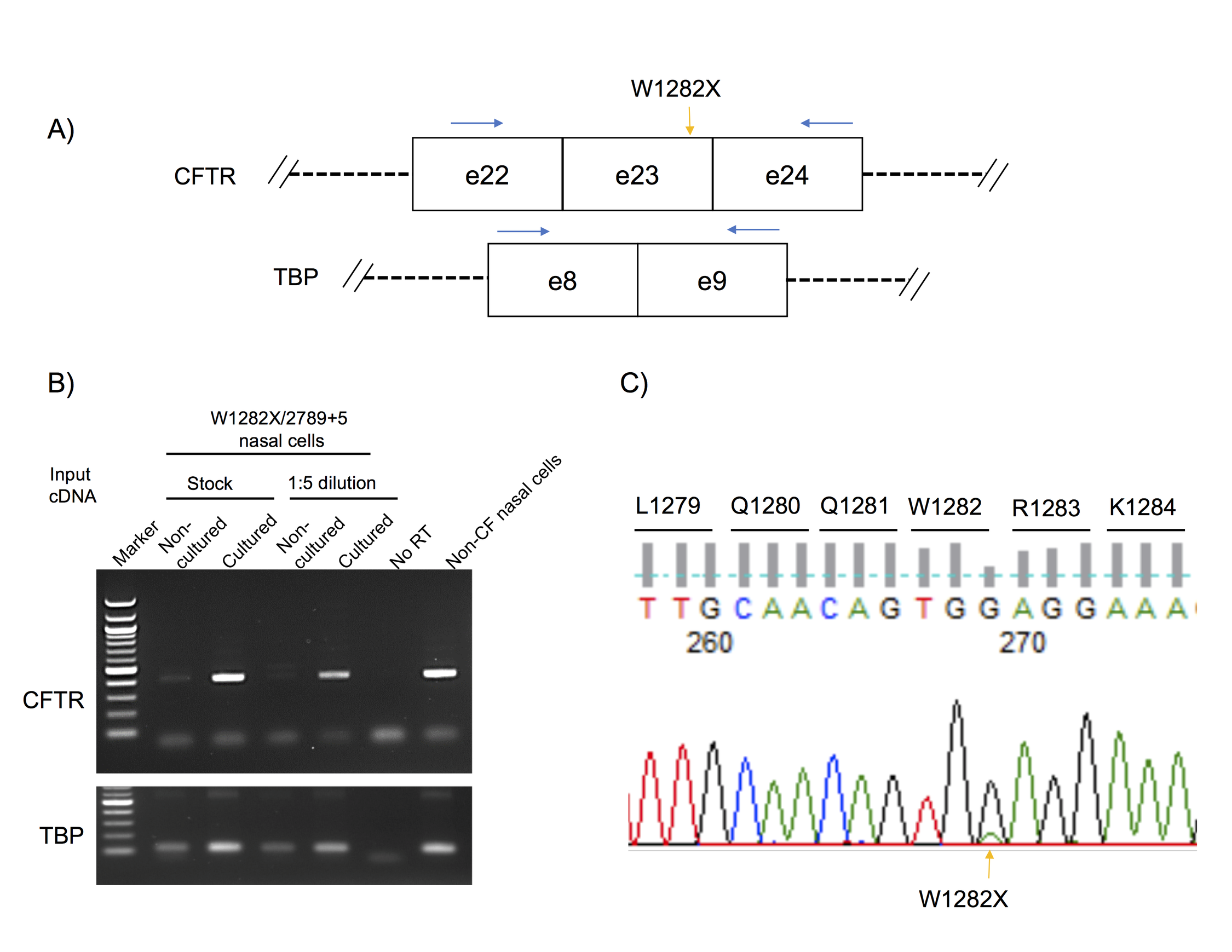

Supplement: S7 Fig — (A) Top panel, schematic illustration of the region selected to amplify CFTR. Vertical arrow indicates location of W1282X variant in the context of processed mRNA. Horizontal arrows indicate forward and reverse primers selected from exon 22 and exon 24 respectively for the reverse transcription-polymerase chain reaction (RT-PCR). Bottom panel, shows selection of primers to amplify TATA box binding protein (TBP) gene as control. (B) Ethidium bromide—stained agarose gel to visualize RT-PCR products. RNA was extracted either directly from the nasal cells dislodged from brush using a forcep (non-cultured) or nasal cells expanded in propagation medium containing 10 μM reagent-Y (see methods for culture and S3 Table for recipes). 200 ng RNA was used to prepare cDNA. PCR was performed on either stock cDNA or diluted (1:5). cDNA prepared from the non-CF cultured nasal cells was used as a positive control. The molecular weight of the amplification product matched the expected size products for CFTR (417 base pairs) and TBP (108 base pairs). Faint amplification was achieved for stock cDNA prepared from the brush, and very faint amplification for 1:5 diluted cDNA. TATA box binding protein (TBP) was amplified as control for the quality of RNA. No-RT, used as negative control, contained RNA from the cultured nasal cells of individual with 2789+5/W1282X. (C) Representative electropherogram of the RT-PCR product to assess differential expression of mRNA produced from 2789+5 and W1282X alleles. RT-PCR products obtained were sent for Sanger sequencing. Small peak for “nucleotide A” (indicated by vertical orange arrow) corresponding to W1282X allele, and large for “nucleotide G” corresponding to normal spliced wild-type mRNA produced from 2789+5 were observed. RT-PCR result shown here lends support to RNA-seq data that 2789+5 results in partial missplicing, and corroborates with residual CFTR function in the nasal cells observed by short-circuit current measurements. (TIF) [file pgen.1009100.s007.tif]

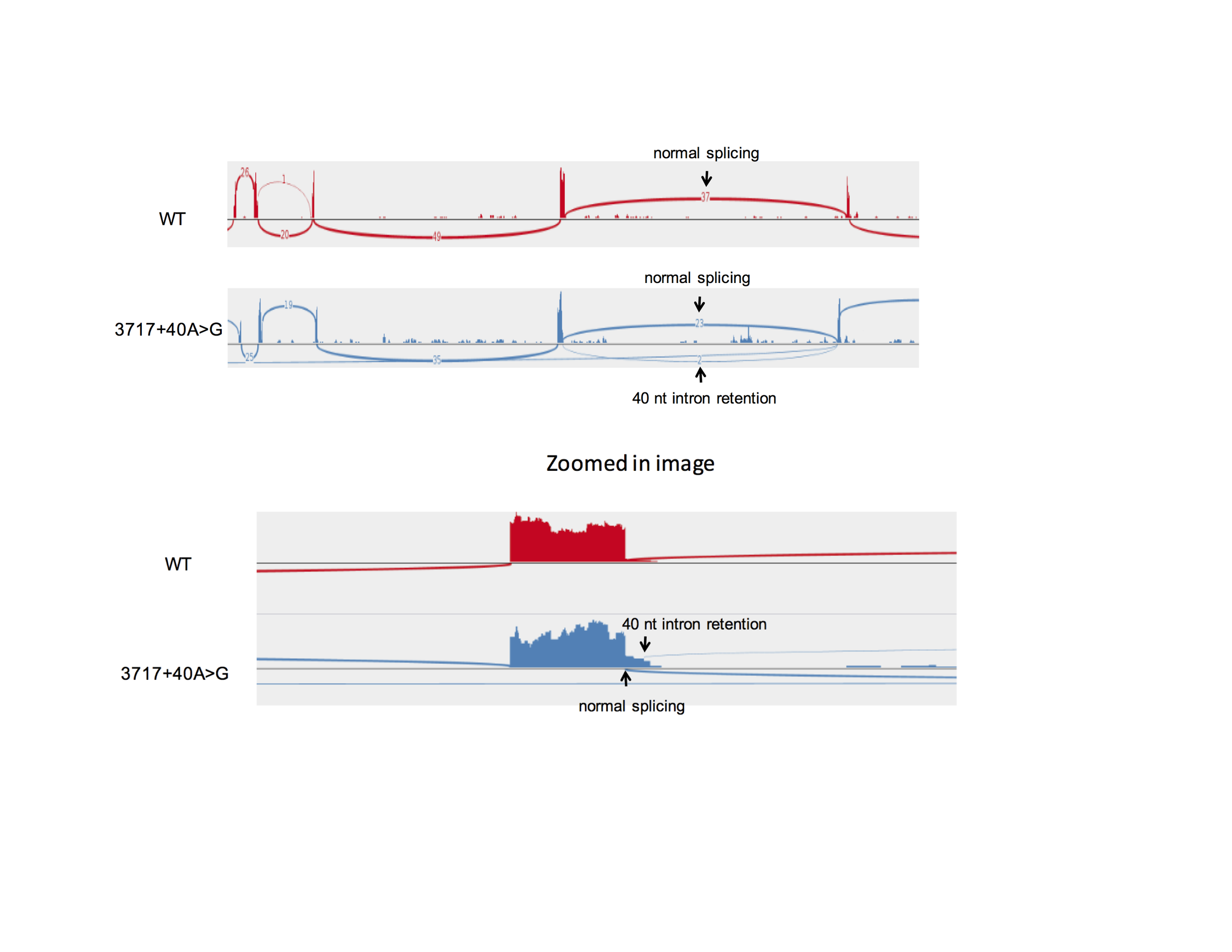

Supplement: S8 Fig — Top panel. Sashimi plot shows retention of 40 nucleotides of intron 23 as well as normal splicing. WT HNEs served as control. Numbers indicate number of reads mapping to each splice isoform. Bottom panel. Zoomed in version of sashimi plot for better visualization of intron retention. (TIF) [file pgen.1009100.s008.tif]

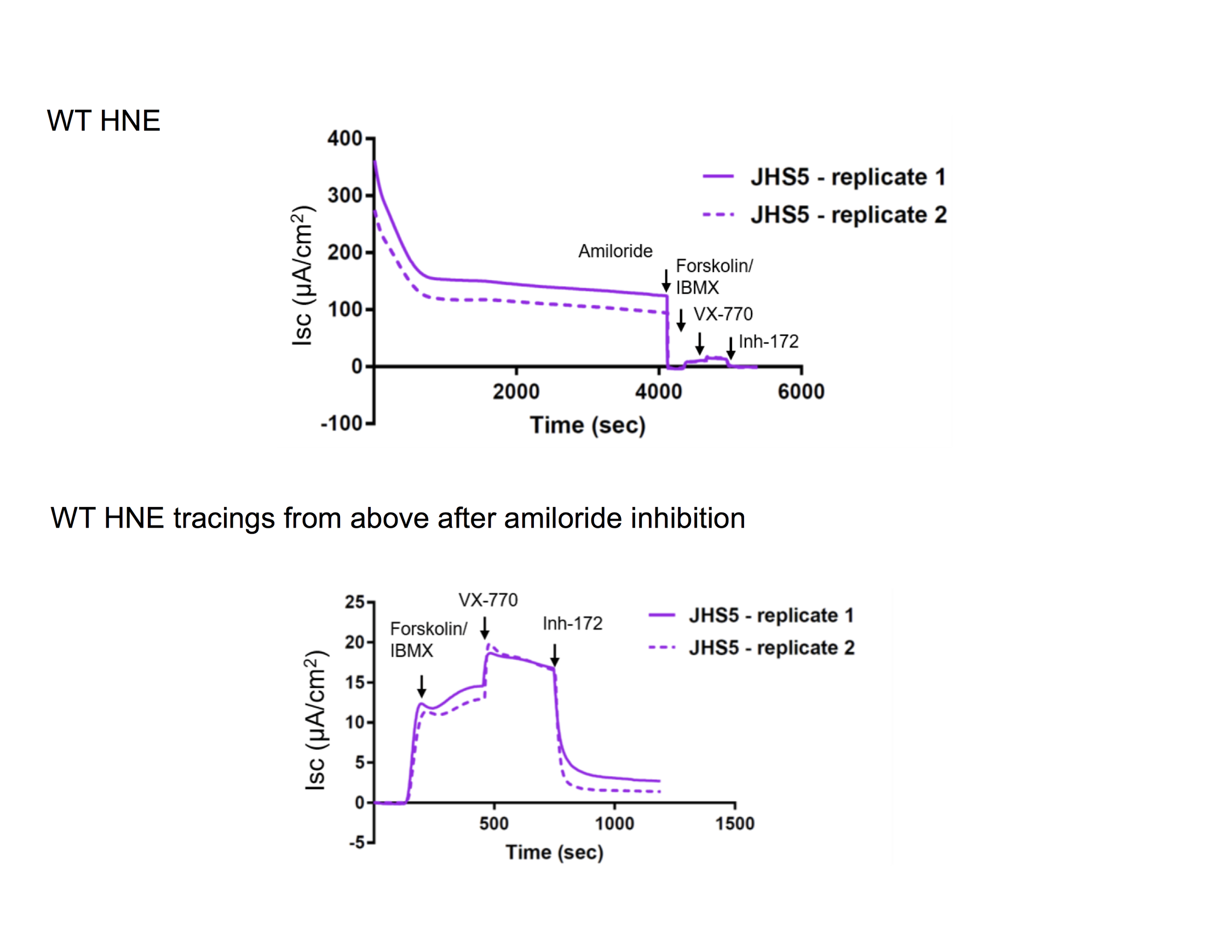

Supplement: S9 Fig — Upper graph, two replicates of short-circuit current (Isc)tracings from the same sample. Lower graph, same Isc tracings as above, but after amiloride inhibition was set to zero to allow for better visualization of the function. (TIF) [file pgen.1009100.s009.tif]

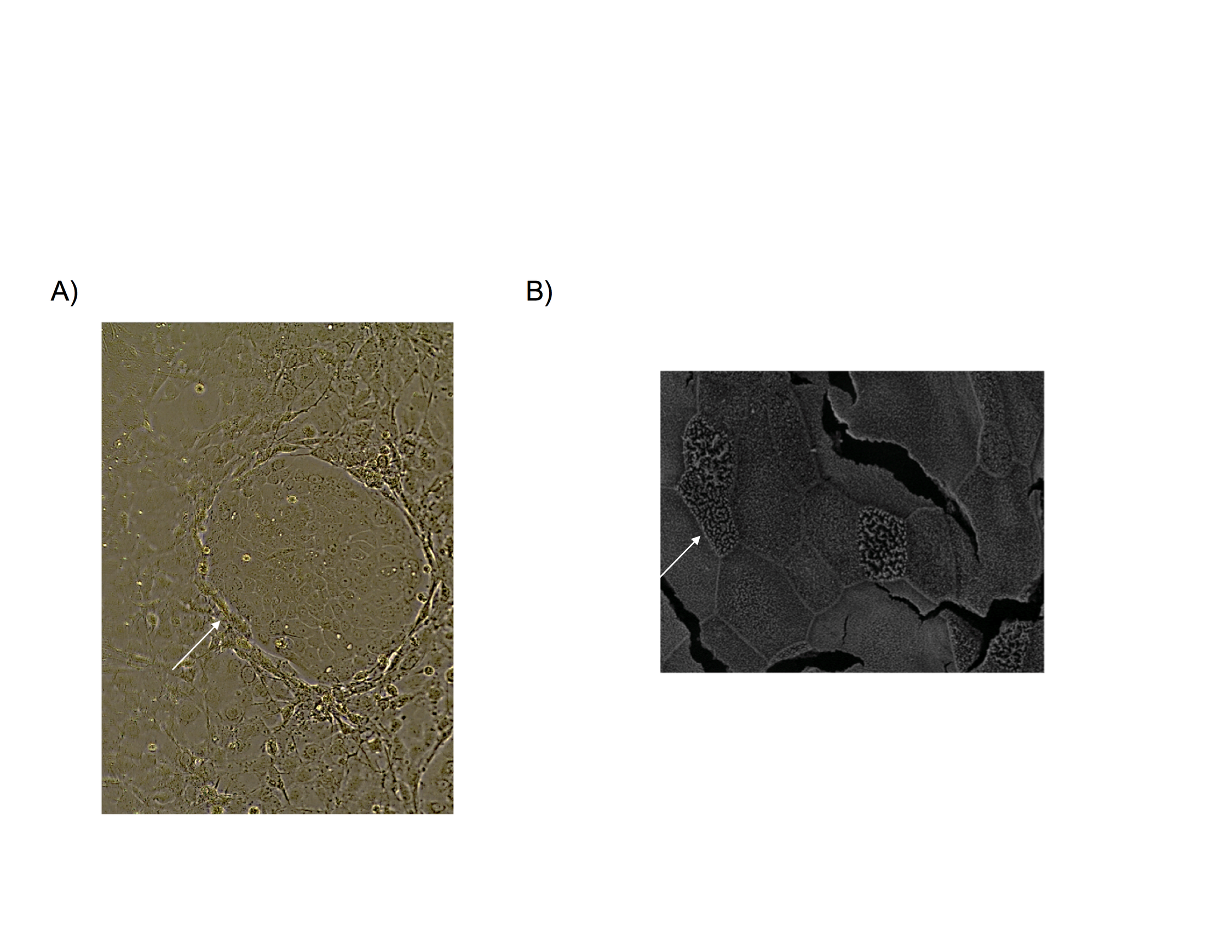

Supplement: S10 Fig — (A) Compound microscopy image of HNE cells propagating in conditionally reprogrammed co-culture with irradiated J2 feeder cell (J2s) at passage 0. Arrow indicates HNE cells growing in island surrounded by J2 fibroblast. (B) Scanning electron microscopy image of well- differentiated 28-days old HNE cells growing in air-liquid interface (ALI) culture. Cells propagating at passage 2 were transferred on to the filters to establish ALI. Arrow indicates appearance of cilia as a marker of differentiation. (TIF) [file pgen.1009100.s010.tif]

c.166 G>A (E56K)

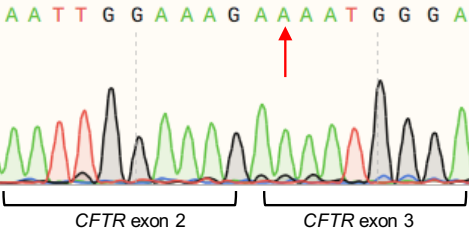

c.581 G>T (G194V)

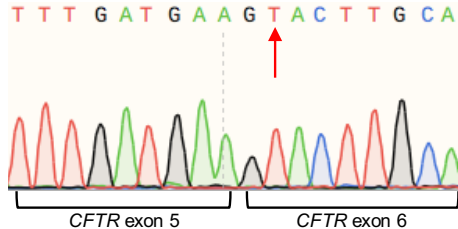

c.580 G>A (G194R)

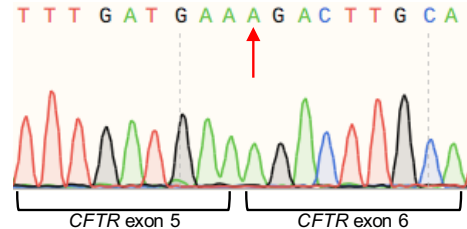

c.3719 T>G (V1240G)

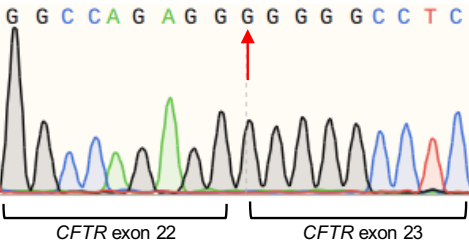

c.3872 A>G (Q1291R)

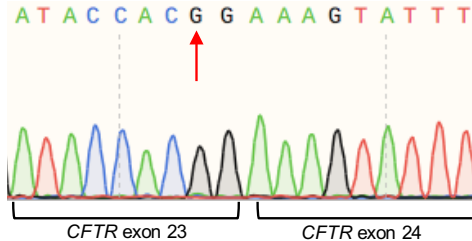

Supplement: S1 Data — (PDF) [file pgen.1009100.s014.pdf]
