# Supplementary material for: Evaluation of both exonic and intronic variants for effects on RNA splicing allows for accurate assessment of the effectiveness of precision therapies
Source: PLoS Genet. 2020 Oct 21;16(10):e1009100. doi: 10.1371/journal.pgen.1009100 (PMC7605713; doi:10.1371/journal.pgen.1009100)
Supplement: S2 Data — (PDF) [file pgen.1009100.s015.pdf]

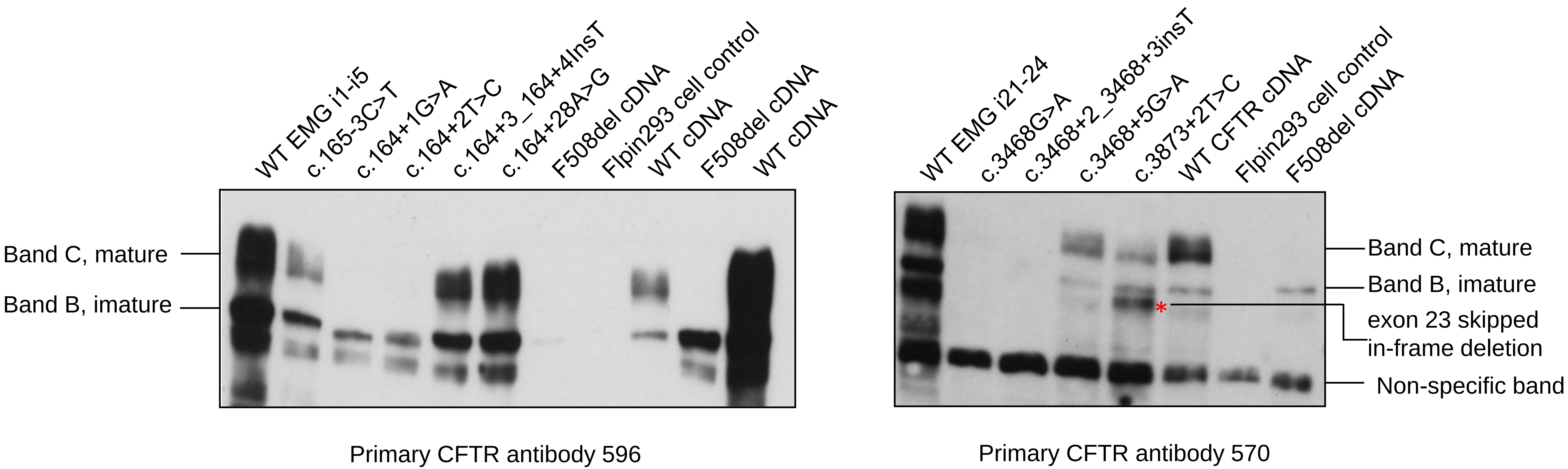

WT EMG i1-i5
c.165-3C>T
c.164+1G>A
c.164+2T>C
c.164+3_164+4InsT
c.164+28A>G
F508del cDNA
Flpin293 cell control
WT cDNA
F508del cDNA
WT cDNA
Band C, mature
Band B, imature
Primary CFTR antibody 596
WT EMG i21-24
c.3468G>A
c.3468+2_3468+3insT
c.3468+5G>A
c.3873+2T>C
WT CFTR cDNA
Flpin293 cell control
F508del cDNA
Band C, mature
Band B, imature
*
exon 23 skipped
in-frame deletion
Non-specific band
Primary CFTR antibody 570

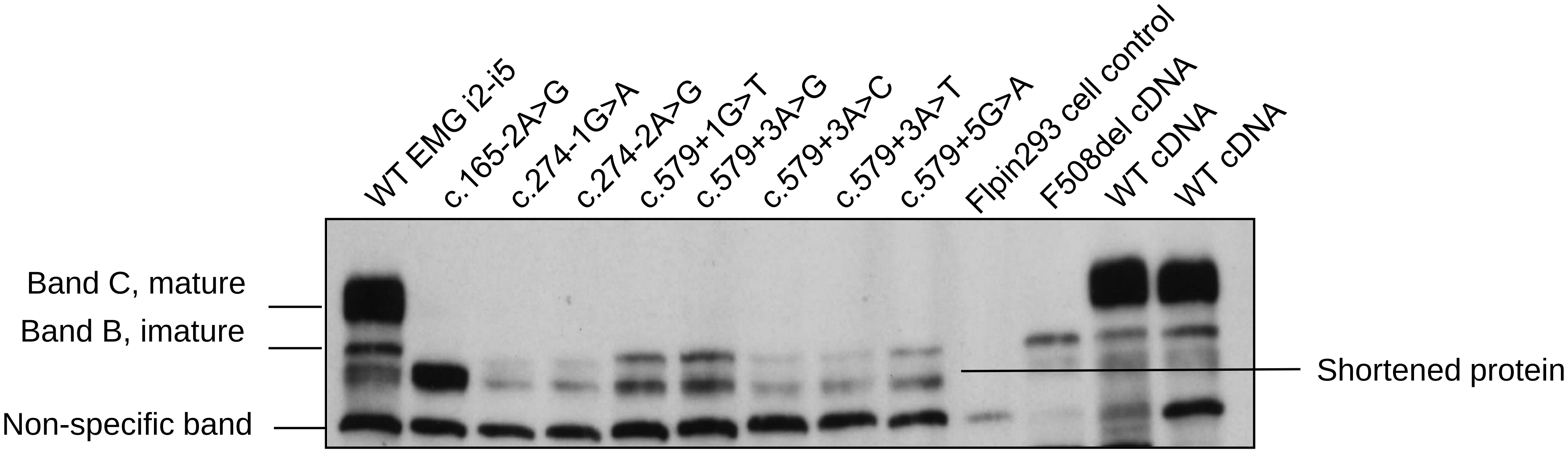

Primary CFTR antibody 570

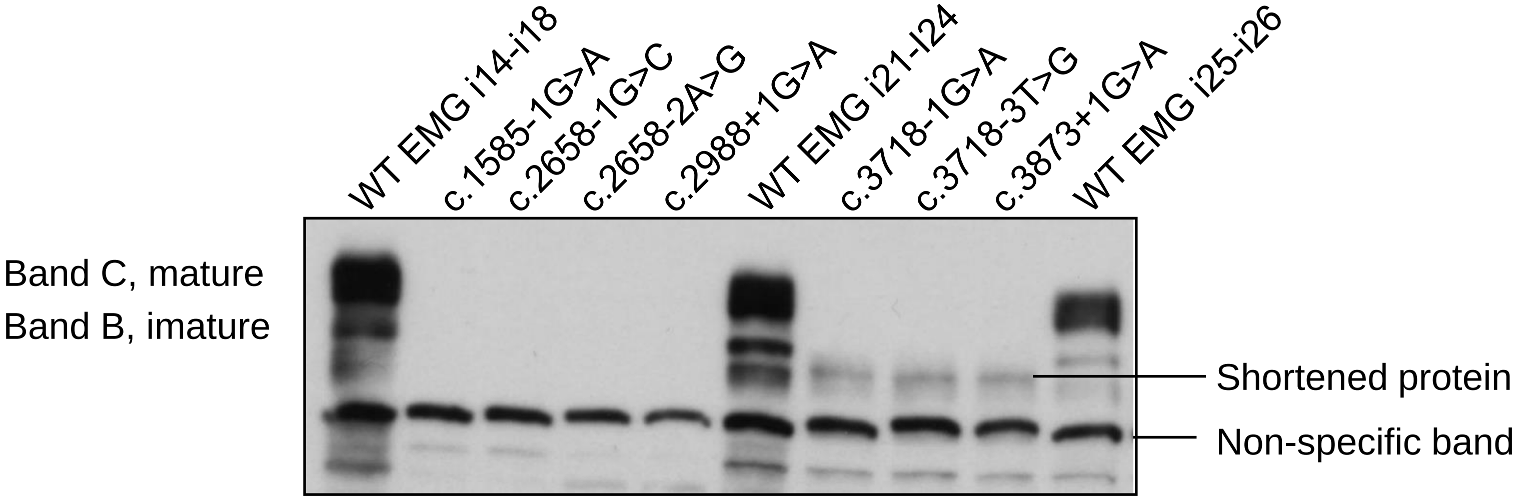

Primary CFTR antibody 570
