# Supplementary material for: Evaluation of both exonic and intronic variants for effects on RNA splicing allows for accurate assessment of the effectiveness of precision therapies
Source: PLoS Genet. 2020 Oct 21;16(10):e1009100. doi: 10.1371/journal.pgen.1009100 (PMC7605713; doi:10.1371/journal.pgen.1009100)
Supplement: Attachment — Submitted filename: splicepaper_responsetoreviewers_final.docx [file pgen.1009100.s020.docx]

Reviewer's Responses to Questions

**Comments to the Authors:**

Reviewer #1

We thank the reviewer for their positive feedback and nice summary of our work.

Reviewer #2:

The authors could broaden the interest by emphasizing the carful molecular assessment (i.e. splicing) of all variants is necessary as more diseases move into genotype driven precision therapies.

>We thank the reviewer for their feedback. We have updated the text to broaden the implications of our work beyond CF (see lines 519-522 (pg24), 600-606 (pg28)).

Minor Concerns

1. At various points it is very hard to figure out what they did and to how many variants. The authors should use consistent language, for example they switch from exonic, to missense to “variants of interest” within the same paragraphs on page 7 and 8. As I believe all variants tested are of interest, and exonic ones tested are missense, while all intronic ones are not, I would prefer they simplify and use “exonic” or “intronic” unless necessary to denote a functional consequence they uncover.

>We agree and have updated the text to use more consistent and clear language when referring to each category of variants.

2. More description or details on CF for those not PLOS Genetics readers well versed in the disease. For example on #285 “We were interested in this variant due to its varying clinical consequences” they can describe the varying clinical consequences, which are described only as a broad sweat chloride range on line #511.

>We have updated the text to provide more details about the clinical presentation of CF (see lines 79-85 (pg4)).

3. Fig 3B, Add data for WT CFTR so we can see how close is Luma+Iva to WT CFTR. The text states “approximately 80% of WT function” for Gln1291His, but this is not cited in any way and it’s unclear if this is a protein level/single channel measurement, or a measurement from cells genetically homozygous for 3873G>C.

>We thank the reviewer for noting this issue. The function of CFTR bearing Gln1291His is estimated to be equivalent to 80% of WT based on measurements of WT CFTR using the same method and cell types. The method for normalization was previously described in Raraigh et al, AJHG 2018 (PMID:29805046). We have updated the text to include this information (see lines 312-313 (pg14)). We have not added WT CFTR data to Fig 3B as this data is previously published.

4. Figure 4 showing data in HBE cells from a patient is a nice validation of what they see in EMG assays. I know procuring patient samples for rare genotypes is difficult, but any additional assays in primary cells would greatly strengthen this papers reliance on the EMG assays.

>We agree. As noted, obtaining primary cells bearing rare genetic variants is challenging and even more so during the current pandemic. While we have included all of the primary cell data that we have been able to obtain, we have updated the text to include additional published studies (see lines 459-462 (pg22)) reporting CFTR splicing isoforms detected in primary airway cells bearing CFTR variants we have studied using the EMG assay. As noted, our EMG results accurately reflect splicing patterns observed in the primary airway cells

Reviewer #3: Comments to Author:

1. The use of expression minigenes (EMGs) and heterologous expression in HEK cells are outdated technologies with known artifacts. This type of study should have been done with CRISPR/Cas9 modification of the endogenous locus in airway and intestinal cell lines at minimum, since primary epithelial cells are rather difficult to engineer or, in iPSCs with subsequent differentiation in to relevant epithelial cells.

>We suggest that all cell-based representations of *in vivo* consequences have limitations. Generation of cell lines using CRISPR/Cas9 technology is labor intensive and efficiency varies greatly between targets, as evidenced by the small number of variants introduced to *CFTR* to date by genome engineering (Valley et al, JCF 2019PMID:30563749, Jennings et al, JCF 2019 PMID:**30670178**). Genome editing can also have off target effects that may alter the function of genes relevant to transcription, splicing or RNA degradation. In addition, a suitable airway epithelial cell line that consistently expresses *CFTR* is not readily available or has additional problems. For example, a widely used bronchial epithelial cell line derived from a healthy individual (16HBE14o-) has endogenous aberrant *CFTR* transcripts, as demonstrated by Valley et al (PMID: 30563749). We have corroborated these results (See left figure below). We also considered a well-established tracheal epithelial cell line, but found that it did not produce detectable CFTR by western blot (See right figure below). Calu-3 cells are another cell line used for such experiments, however they are known to have extensive aneuploidy, which complicates gene editing experiments.

Fig. RT-PCR performed on cDNA from 16HBE14o- cells. Each lane shows products from a different RT-PCR using primers spanning the indicated *CFTR* exons. Red arrows indicate aberrant transcripts.

Fig. Immunoblot showing HTE cells compared to HEK293s transiently expressing WT cDNA CFTR or F508del cDNA CFTR.

>While we agree that iPSCs are a useful tool for studying variants of interest in the native genomic context and cell type, we believe that this method would not allow us to perform the breadth and depth of experiments that we have here due to the sensitive nature of such cells.

>We therefore present the EMG system as a reasonable approach to evaluate the splicing effects of many DNA variants. In response to the reviewer’s concerns, we have added a more detailed discussion of the limitations of expression minigenes to the text (see lines 448-462 (pg21-22)). We have also distinguished the EMG assay from hybrid minigenes, as the latter contain only minimal context from the splice site under evaluation. In contrast, EMGs contain either entire introns or at least 200 bp of flanking introns and encompass multiple sequential exons and introns. Consequently, as more complete representations of the splicing environment, EMGs demonstrate a high degree of fidelity with *in vivo* splicing patterns (see lines 460-462 (pg22)).

2. The authors have ignored an extensive literature on complex intronic and exonic elements that impact spicing, and on the fundamental role of chromatin features on splicing and RNA processing.

>We mention intronic and exonic splice elements in the original mansucript (see lines 405-408) but have revised the text to address the reviewers concerns by broadening our discussion to include chromatin-regulation as well as add additional references (see lines 420-425 (pg 20)). We agree that transiently expressed EMGs that are episomal cannot replicate chromatin-level impacts on splicing. However, stable integration of EMGs into the genome of CFBE cells could allow for chromatin-level regulation, albeit at a different genomic locus than the endogenous *CFTR* gene.

3. To define variants as "deep" intronic (eg Table 2) when they are 20 or 40 bp away from the splice site is erroneous. Many CFTR introns are greater than 10 kb long and it is known that there are well defined regulatory elements and splicing variants truly "deep" within these introns, several kb away from intron/exon boundaries. The variants described in this manuscript all lie within an extended splice site machinery.

>We agree. We know that there are truly ‘deep’ intronic variants (e.g. 3849+10kb), and wish to distinguish between variants within the nucleotides that directly interact with the spliceosome and those that are in the extended splice regulatory region. We have updated Table 2 and the text so that the groupings are more clearly defined by the distance relative to the exon/intron boundary. We now refer to those variants in the +3, +4, +5, and -3 positions as “proximal intronic” and those outside of the consensus splice site as “distal intronic” (see lines 333-335 (pg15)).

4. How is the fragment analysis quantified in SFig 2, F2A, F3A, etc? It would seem difficult to reliably quantify PCR amplicons that are gel extracted prior to fragment analysis. The recovery efficiency of gel extraction is very low and variable from extraction to extraction. This could influence the quantification of transcripts derived from the same EMG (as presented in the mentioned figures).

>We appreciate the reviewer noting this technical limitation. We want to clarify that our gel extractions were performed in triplicate in order to account for variability in technical efficiency. The relative transcript quantities obtained from each extraction were averaged to give the final percentage and the variation is reflected in the error bars in Fig 2A and Fig 3A. We have updated the text of the methods to clarify this point (see lines 646-647 (pg 30)) and we have added the following description explaining the quantification: “Relative RNA isoform quantity was determined by the area under the curve (AUC) for each isoform compared to the total AUC for both isoforms for each sample and these values were then averaged across the technical replicates” (see lines 650-652 (pg30))

5. If isoform 2 of c.2908 variants produces S912-G970 deletion (Figure 2A, left), and c.2908G>A produces more of this transcript than isoform 1, how can the authors be confident that the protein species detected for the EMG constructs of the two variants that cause missplicing are from isoform 1 and not isoform 2? The predicted protein sizes are not given, nor are any molecular weight markers noted on the western blot in Figure 2B. To better estimate the identity of this protein species, the authors should express the cDNA of these predicted protein species and compare to lanes 5 and 7 in the current western blot.

>We appreciate the reviewer’s careful observation. We have revised Fig 2B to show the molecular mass of this species as well as the expected molecular mass of band B and band C. We have also edited the text to explain that we interpret the shortened protein observed in Fig 2B is the result of isoform 2, as isoform 1 would be expected to undergo nonsense-mediated mRNA decay. Furthermore, even if stable truncated protein was produced, it would not be detected due to the location of the antibody binding downstream of residue 970 (see lines 208-213 (pg10), 226-230 (pg10-11)). Additionally, we identified an error in the labeling in Fig 2A that may have contributed to the appearance of incongruence between mRNA and protein results and have corrected this error as well as the corresponding text.

6. The conclusion made on lines 240-245 are not fully supported by the data presented. cDNA constructs were not made that would generate the predicted protein sequence of the misspliced c.2909 variants, and tested in short circuit current studies, therefore, it is only speculation that these immature protein species would be non-functional.

>While we did not test the function of the protein expected to be produced by c.2908G>A and c.2908G>C, a prior publication reported that individuals bearing c.2908G>C do not respond to modulator therapy (De Boeck, JCF 2014 PMID: 25266159). Given that we have shown that c.2908G>A produces the same two RNA isoforms as c.2908G>C, we think it is reasonable to conclude that c.2908G>A would also fail to respond. However, we appreciate the reviewer’s point and we have reworded these lines to make it clear that this is an inference supported in part by our data and in part by the reference given here (see lines 253-256 (pg12)).

7. How does the short circuit current experiments for the stable lines in CFBE compare to the parental line (CFBE with Flp integration)? That is, how can the change in channel activity be attributed to the integrated cDNA construct and not to the effect of the drugs on the F508del proteins in these cells (CFBE/CFBE41o- is F508del homozygous), especially since Orkambi (lum/iva) can be prescribed for and ivacaftor has been reported to affect channel activity of F508del in cultured HBE cells (Van Goor, PNAS 2009, PMID: 19846789). The contribution of F508del to the signal is mentioned in the description of the results of figure 4C (pg 18), however again, it is unclear how the authors can conclude with the data presented, that the responses of the cells in figure 4C, especially in regards to ivacaftor alone, are contributed by the 3717+40G>A allele and not F508del.

>We thank the reviewer for noting this point. While CFBE41o- and thus our derivative CF8Flp cell line does have an F508del/F508del genotype, we have previously shown through extensive characterization that the endogenous *CFTR* genes have been silenced and do not produce any detectable *CFTR* transcript, protein, or function (Gottschalk et al, JCF 2016 PMID: 26694805, Supplemental Fig 1, Supplemental Fig 5). While subclones of CFBE41o- cells have been generated after transfection that express F508del CFTR (e.g. CFBE41o-4.7, Illek et al, Cell Physiol Biochem 2008, PMID:18769032), the specific clone of CFBE41o- cells we obtained directly from Dieter Gruenert has no endogenous *CFTR* expressed (Illek et al, Cell Physiol Biochem 2008, Figure 2 “parental”). Other investigators have reported the same finding in CFBE41o- cells provided by Dr. Gruenert (Bebok et al, J Physiol 2005, PMID:16210354, Figure 1). As a result, we are confident that channel function that we detect via short circuit current is the result of expression of our integrated construct only. Additionally, extensive study of a variety of *CFTR* variants in our CF8Flp system has identified variants that are expressed in these cells but show no response to modulator therapies (Han et al, JCI Insight 2018, PMID:**30046002**), indicating that the endogenous CFTR is not producing any modulator response. We have updated the methods to make it clear that our CF8Flp cells originate from CFBE41o- cells that do not have endogenous *CFTR* expression (see lines 670-671 (pg 31)).

>As for 3717+40A>G, we agree that the modulator response in primary cells shown in figure 4C is in part from the F508del allele that is *in trans*. However, the complementary data in figure 4D, in which we expressed 3717+40A>G alone in our CFBE cell line, supports the conclusion that the normally spliced WT transcript produced by 3717+40A>G is modulator responsive. We have expanded upon this point in the text to provide more clarity (see lines 398-399 (pg19)). Additionally, we have added a supplemental figure (**S9 Fig**) showing that WT HNEs respond to ivacaftor treatment. While we agree with the reviewer that we cannot completely rule out some contribution of the F508del allele to the observed ivacaftor response, we note that in the reference the reviewer points to (Van Goor et al, PNAS 2009, PMID:19846789) only 3/6 F508del/F508del HBE cultures showed very modest response to ivacaftor. Additionally, this result is inconsistent with *in vivo* results in which ivacaftor alone yielded no clinical benefit in F508del homozygotes (Flume et al, Chest. 2012, PMID: 22383668). Given the magnitude of the ivacaftor response we observed without use of correctors, we postulate that ivacaftor is acting primarily on residual normally spliced transcript from 3717+40A>G and not F508del-CFTR.

8. Comprehensive raw data is not shown for “RNA effect” and “Protein present” columns for all variants in both tables, this should be provided in some form as supplementary data, and most especially for variants further explored in subsequent figures. It is unclear if protein production was assessed via western for each variant, or if predictions were made based on sequencing of RNA species. Additional information is also important in order to support statements like those made on line 330, in relation to the two intronic variants that express reduced levels of full-length transcript--was that calculated in relation to the WT EMG construct that harbored these mutations? How much reduction of transcript? Was it significantly different? A quantitative assay is needed to support this statement. This would also be helpful to support the conclusion that the “shortened protein isoform produced by this variant (from the EMG of c.454A>G)….” (line 487) is in fact producing a protein species.

>We appreciate this critique and, as recommended, we provide additional data to the supplementary materials for the tables including Sanger sequencing (**S1 Data**) and immunoblots (**S3 Fig**, **S6 Fig, and S2 Data**) to clarify that protein production was assessed by immunoblotting for CFTR and not by predictions (For example, see Fig. below). This figure has been added to supplementary material (**S6 Fig**).

We also refer the reviewer to the references in Table 1 & Table 2, which indicate our previously published data on experimental assessment of splicing effects by 1) Sanger sequencing or fragment analysis of RNA species, and 2) immunoblotting for protein production on a subset of these variants. Please see Fig 3 for variants c.2789+2InsA, c.2789+5G>A in Sharma et al. Hum Mutat 2014 (PMID: 25066652). For the variant c.454A>G please see Fig 3A in Lee et al AJHG 2018 (PMID: 28475858), which supports the results shown in Table 1, as well as Fig 4B for the variant c.3717+40A>G.

**S6 Fig**. Residual normal splicing and protein production resulting from c.4242+2T>C variant in the CFTR terminal intron.

We appreciate the reviewer’s point that “reduced levels of full-length transcript” implies quantification relative to WT EMGs. As our methods did not allow for this normalization, we have revised the text to use the term “residual full-length transcript” to clarify that what we mean is that missplicing is incomplete in these instances and there is some level of normal splicing occurring as well.

9. HNEs with the genotype c2657+5G>A/W1282X are used to assess missplicing caused by the intronic variant, while comparing to a WT control (Figure 4A). The authors conclude that because W1282X is a nonsense variant, the presence of 9 reads that map from E15 to E16 and 3 reads that map from E15 to E17 as compared to the 27 reads in WT mapping from E15 to E16 mean that the intronic variant only causes partial missplicing. However, since W1282X is in exon 23, and is known to produce some detectable levels of mRNA transcript that can be measured under normal conditions (for example: Linde et al JCI, 2007, PMID: 17290305), the authors should use a W1282X homozygous HNE sample for control to fully conclude that the normal splicing detected between E15/E16 is not contributed by the W1282X allele.

>We agree with the reviewer’s point that W1282X mRNA levels are variable. We refer the reviewer to a prior publication (Aksit et al, JCF 2019 Fig1, PMID:30803905) in which we demonstrate that W1282X mRNA undergoes efficient nonsense-mediated mRNA decay by studying W1282X carrier parents because their wild type CFTR allele serves as an internal control and also show bulk RNA-seq results from the primary nasal cells in a W1282X homozygous individual to compare with non-CF healthy individual and carrier parents (Fig2). Additionally, we have performed additional experimentation with RT-PCR and Sanger sequencing of cDNA from the HNEs used in the experiments for Fig 4A and show that the majority of the transcript comes from the c.2657+5G>A allele (see below & **S7 Fig**, see lines 573-579 (pg27)). We also note that the presence residual CFTR function in c.2657+5G>A/W1282X HNEs provides additional evidence that the c.2657+5G>A allele generates some full-length transcript (and hence, full-length protein), as W1282X truncated protein has been shown to be non-functional in primary nasal cells (Haggie et al, JBC 2017, PMID: **27895116**).

**S7 Fig.** Sanger sequencing from RT-PCR performed on c.2657+5G>A/W1282X HNEs. Region shown is *CFTR* exon 23. Orange arrow indicates “A” peak coming from W1282X allele.

Minor comments:

1. Please provide full details of antibodies that were used in this study.

>We have revised the methods to give more detail about the antibodies (see line 658-663 (pg30)).

2. It would be useful to remind readers that the CFBE/FLP cell line only has one copy of the FLP recombinase (reference 29), and that therefore integration copy number should not impact EMG or cDNA expression values between cell lines as all lines should have 1 integration.

>Good point. We have revised the text to note that our cell lines have a single integration site (see lines 670-671 (pg31)).

3. Please provide in the methods summary for “Collection and culture of primary HNEs” additional details on growth conditions (ie media used, disassociation reagent, concentration of ROCK inhibitor, approximately how long cells were cultured at ALI, etc)—having to sift through 4 references is tedious.

>We have revised the methods section to provide more detail on the culture of primary cells (see lines 684-705 (pg32)) and we have added supplementary materials including formulas for culturing media and specific information for all reagents used (**S3 Table**). In addition, we added a compound miscroscopy image of primary nasal cells in propagation phase (media containing ROCK inhibitor) and electron microscopy image of well-differentiated airway liquid interface (ALI) culture (see **S10 Fig**).

4. While the focus of this manuscript is on non-synonymous substitutions in exons, there have been multiple reports of synonymous substitutions that alter CFTR splicing. Reference of these studies could further strengthen the argument to further characterize variants by other means than just predicted protein sequence alterations.

>We appreciate this point. We have added additional sentences to the discussion to address the role of splicing in synonymous variants (see lines 513-522 (pg24)) and refer the reviewer to our previous publication (Sharma et al, Human Mutat 2014, PMID:25066652) in which we identified one such variant.

5. How were the variants indicated in the tables identified? Were these all the variants from the CFTR Mutation Database (Toronto) or from the CFTR2 list? If the latter, what list version was used? (page 6, lines 127-129, page 15 lines 322-323).

>All variants were identified in the CFTR Mutation Database. Variants with an allele count of less than three are not reported in CFTR2. For those variants which meet criteria for inclusion in CFTR2, phenotype data was obtained from the January 10, 2020 update (see line 754-755 (pg35).

6. What is the scale of the y-axis for the fragment analysis shown (SF1)?

>We have updated S1 Fig with a clearer y-axis.

7. Please provide the molecular weight markers for the western blot in Figure 2B, as well as numbering the lanes as described in the text on page 10.

> We have revised Fig 2B with lane numbers and have indicated the molecular mass of the relevant protein isoforms.

8. References for previously reported primer sequences should be provided within the table

>We have revised S2 Table to include references for previously reported primer sequences.

9. For HNE electrophysiology in Figure 4, the results note the % channel function as it corresponds to WT HNE (pg 18), however these traces/values are not provided.

> The percent channel function in HNEs was determined by comparing the ∆Isc value to the average ∆Isc observed in WT HNEs from two different individuals across 11 total recordings (ΔI_sc_ = 14.2μA/cm^2^ ±4.0). We have revised the text to include this information (see lines 392 (pg19), 732-733 (pg34)). Additionally, we have added a representative WT HNE short circuit tracing as **S9 Fig**.
